# Impaired Attention Orienting in Young Children With Fragile X Syndrome

**DOI:** 10.3389/fpsyg.2019.01567

**Published:** 2019-07-10

**Authors:** Mariya Chernenok, Jessica L. Burris, Emily Owen, Susan M. Rivera

**Affiliations:** ^1^ Department of Human Ecology, University of California, Davis, Davis, CA, United States; ^2^ Center for Mind and Brain, University of California, Davis, Davis, CA, United States; ^3^ Department of Psychology, Rutgers University, Newark, NJ, United States; ^4^ Department of Psychology, University of California, Davis, Davis, CA, United States; ^5^ MIND Institute, University of California Davis Medical Center, Sacramento, CA, United States

**Keywords:** fragile X syndrome, gap-overlap paradigm, attention shifting, eye tracking, attention orienting

## Abstract

Fragile X syndrome (FXS) is a genetic disorder caused by a trinucleotide CGG expansion within the *FMR1* gene located on the X chromosome. Children with FXS have been shown to be impaired in dynamic visual attention processing. A key component of dynamic processing is orienting—a perceptual ability that requires disengagement and engagement of attention from one stimulus to fixate on a second. Orienting, specifically the disengagement and engagement of attention, has previously not been studied in young children with FXS. Using an eye tracking gap-overlap task, the present study investigated visual disengagement and engagement in young children with FXS, compared to mental age (MA)- and chronological age (CA)-matched typically developing children. On gap trials, the central stimulus elicited fixation, but then disappeared before the peripheral target appeared, imposing a visual gap between stimuli. On overlap trials, the central stimulus elicited fixation, and remained present when the peripheral target appeared, creating visual competition. A gap effect emerges when latencies to shift to the peripheral target are longer in overlap versus gap conditions, reflecting the recruitment of cortical and subcortical disengagement and engagement mechanisms. The gap effect was measured as the latency to orient attention to the peripheral target during gap versus overlap conditions. Both MA and CA groups showed the expected gap effect, where children were slower to orient to peripheral targets on overlap trials than on gap trials. In contrast, in the FXS group, saccadic latencies between gap and overlap trials were not significantly different, indicating no significant gap effect. These findings suggest disrupted attentional engagement patterns in FXS that may be underlying impairments in attention orienting, and suggest potential targets for attention training in this population.

Fragile X syndrome (FXS) is the most common heritable form of intellectual disability with an estimated prevalence of the full mutation of about 1:4,000–7,000 in males and 1:4,000–11,000 in females (Tassone et al., 2012; Hunter et al., 2014). FXS is a genetic disorder caused by an unstable expansion of the CGG trinucleotide within the *FMR1* gene. An expansion that exceeds 200 CGG repeats results in methylation or shutting down of the production of the critically important fragile X mental retardation protein (FMRP) (Coffee et al., 2009; Hagerman et al., 2017). FMRP is essential for healthy dendritic translation and synaptic plasticity in the brain, and a reduction of this protein negatively impacts brain and cognitive development (Bassell and Warren, 2008; Kim et al., 2012; Hall et al., 2016). Individuals with FXS are characterized by mild to severe intellectual disability and high rates of comorbid diagnoses of autism spectrum disorder (25–60%), anxiety disorders (70–83%), and attention deficit/hyperactivity disorder (30–66%) (Cordeiro et al., 2011; Ciaccio et al., 2017; Kaufmann et al., 2017).

The most severe deficits consistently documented in FXS reflect parietal processes, including visuospatial ability, processing of sequential information, and attentional skills (Munir et al., 2000; Wilding et al., 2002; Loesch et al., 2003; Kogan et al., 2004; Cornish et al., 2012; Huddleston et al., 2014; Del Hoyo Soriano et al., 2018; Hooper et al., 2018).

## Development of Visual Attention

A key component of dynamic visual attention is visual orienting, the shifting of attention to fixate to a stimulus. Flexible and efficient attention shifting is essential for selecting and processing information in the environment. This skill typically emerges relatively early in infancy and is supported by a distributed fronto-parietal attention network, including the subcortical superior colliculus, frontal eye fields involved in voluntary saccades, prefrontal cortex involved in inhibiting unwanted saccades, and dorsal regions of the parietal cortex (Csibra et al., 1998; Shomstein, 2012; Kulke et al., 2017).

At birth, a typically developing infant is only capable of reactive orienting, a comparatively low-level stimulus-driven (i.e., reflexive) behavior, supported by subcortical structures like the superior colliculus in the midbrain (Johnson, 1990). However, during the first 6 postnatal months of typical development, rapid synaptogenesis and reorganization in the frontal and parietal cortex result in increased cortical control over subcortical regions (Farroni et al., 1999; Kulke et al., 2017). This cortical network modulates the lower level pathways that function from birth, facilitating more efficient and flexible orienting of attention (Johnson, 1990). Thus, when the visual system is engaged, reflexive orienting (*via* subcortical collicular activity) is inhibited in combination by the posterior parietal network, prefrontal cortex, and frontal eye fields, resulting in slower saccadic reaction times (i.e., visual orienting) (Hood and Atkinson, 1993; Csibra et al., 1997, 1998; Shomstein, 2012). However, when attention is disengaged (from a stimulus), reflexive orienting is uninhibited, resulting in faster saccadic reaction times (Fischer and Weber, 1993). Proper development and functioning of these two systems represent the basic building blocks necessary for efficient engagement and disengagement of visual attention. Additionally, neuroimaging studies suggest the role of the parietal cortex in top-down and bottom-up visual orienting involving the dorsal and ventral streams (see Corbetta and Shulman, 2002; Vossel et al., 2014 for review). Importantly, the dorsal visual stream, a pathway extending from the primary visual cortex to the posterior parietal cortex (including activation in both the inferior parietal lobule and superior parietal lobule), carries dynamic spatiotemporal information necessary for motion perception and complex planning of eye movements (Shomstein, 2012). Strikingly, these specific cognitive skills fall under the clinical cluster of dynamic attention deficits documented in infants and toddlers with FXS (Scerif et al., 2005; Farzin and Rivera, 2010; Farzin et al., 2011).

## Visual Attention in Fragile X Syndrome

In FXS, there is considerable evidence that basic visuospatial and visual attention deficits are observable early in development (Scerif et al., 2005; Farzin et al., 2008, 2011; Farzin and Rivera, 2010). Studies have also demonstrated that infants with FXS are impaired on attention-mediated tasks, demonstrating significant deficits in visual motion processing and multiple object tracking (Scerif et al., 2005; Farzin et al., 2008; Farzin and Rivera, 2010). In one study, infants with FXS had a significantly higher detection threshold for dynamic stimuli than for static stimuli, and this difference was not present in a typically developing control group (Farzin et al., 2008). Additionally, infants with FXS demonstrated the ability to encode the location of two static objects over the course of a brief occlusion, but failed to do so when the objects moved during the occlusion period (Farzin and Rivera, 2010). Thus, the visuospatial deficits observed in FXS tend to be those involving parietally mediated attention processing. Further, recent evidence from a *FMR1*-knockout mouse model of FXS suggests that FMRP expression is critical to visual circuit organization and function in the superior colliculus (Kay et al., 2018), a subcortical structure essential to orienting. Given the protracted neurodevelopmental course of the parietal cortex, which undergoes several periods of functional reorganization over the first years of childhood and adulthood, this region is consequently a good candidate area for early disruption to processing and altered cortical network communication that could drive cascading developmental effects (Sowell et al., 2003; Bunge and Wright, 2007).

There is now an extensive literature providing evidence that infants with FXS display differences in visual attention, and that adults on the FX spectrum present with higher level behavioral and cognitive deficits that reflect impairment in selectively parietal, attention-mediated processes (Rivera et al., 2002; Kim et al., 2014). By considering the typical course of neurocognitive development of such processes, we begin to consider that the hallmark cognitive impairments documented in adults with FXS have neurodevelopmental origins in parietal regions that are also associated with the basic deficits observable in infants with FXS. However, more research is still needed to characterize the impact of FXS on visual engagement and disengagement in the context of attention orienting. To date, there is only one report of attention orienting measured by the gap-overlap paradigm in FXS. This study found that adolescent females with FXS, relative to typically developing controls, exhibited slower saccades on overlap trials (reflecting disrupted disengagement), had more difficulty generating predictive saccades, and memory-guided saccades (Lasker et al., 2007). Importantly, females typically present with a milder form of the disorder, due to the normal functioning *FMR1* allele that produces FMRP on the second X chromosome (Huddleston et al., 2014). Thus, additional research is needed with males to examine attention orienting difficulties in more severe forms of the disorder. Further, Lasker et al. (2007) assessed females with FXS ranging from 7.5 to 22.1 years of age (mean age 14.6) while our study includes both males and females ranging from 7.25 to 68.02 months (mean age 39 months). Given the developmental time course of visual attention, evaluating young children with FXS is essential to delineating the impact of the disorder on the developmental trajectory of visual orienting. Accordingly, the current study aimed to investigate attention orienting, specifically disengagement and engagement of attention, in young children with FXS.

## Gap-Overlap Task

The gap-overlap paradigm makes use of two conditions under which attention is shifted from a central stimulus to a peripheral target. In the “gap” condition, a central stimulus elicits fixation (i.e., engagement of attention), but then disappears before a peripheral stimulus appears; there is a visual gap imposed between stimuli. In this condition, extinguishing of attention is automatically induced by the removal of the central stimulus; there is no competition between the stimuli, so orienting to fixate on the peripheral stimulus is automatic and results in faster saccadic response (Hood and Atkinson, 1993). In the “overlap” condition, the central stimulus elicits fixation and then remains present when the peripheral stimulus appears. Therefore, the two stimuli compete for attention, resulting in decreased saccadic reaction times as the viewer must actively disengage from the central stimulus in order to orient toward the peripheral one (Farroni et al., 1999).

When participants are tested on both conditions, a “gap effect” emerges, such that latencies to shift are longer in the overlap condition due to an “engaged” attentional system. This gap effect is interpreted as reflecting the strength of engagement of attention, with recruitment of disengagement and engagement mechanisms. Attention to the peripheral stimulus on gap conditions relies on subcortical maturation, specifically the pathway from the retina to the superior colliculus, whereas on overlap conditions when the visual system is engaged by two competing stimuli, posterior parietal network, prefrontal cortex, and frontal eye fields are necessary for disengagement from central stimulus to the peripheral target (Hood and Atkinson, 1993; Matsuzawa and Shimojo, 1997; Csibra et al., 1998; Farroni et al., 1999). Importantly, connectivity between subcortical and cortical pathways involved in visual processing is necessary for inhibiting reflexive saccades, and orienting of attention (Johnson, 1990; Hood and Atkinson, 1993). Given the evidence to suggest that visual circuits including superior colliculus and posterior parietal networks are impacted in FXS, the gap-overlap paradigm is ideal for studying disengagement and engagement of attention.

## The Current Study

To further investigate the neurodevelopmental impact of parietal dysfunction on visual orienting in FXS, the present study used a gap-overlap task in young children with FXS, as compared to typically developing children. Specifically, we used infrared eye tracking to measure the gap effect, an index of visual orienting efficiency. We predicted that given the known parietal attention deficits in FXS, young children with FXS would not show a typical gap effect, suggesting reduced attentional engagement, relative to typically developing chronological and mental age-matched groups. Based on prior literature showing decreased saccade latencies and increased disengagement abilities with age, we expected that the younger children (mental age-matched group) would show a larger gap effect (appearing as longer latencies on overlap trials, reflecting slower disengagement), relative to the older children (chronological age-matched group).

## Materials and Methods

### Participants

Twenty-four participants with FXS were recruited through the University of California, Davis MIND Institute Fragile X Research and Treatment Center, where they were clinically evaluated, and diagnoses were confirmed by DNA testing. FXS allele status was confirmed by *FMR1* DNA testing and the sample consisted of 18 individuals with the full mutation (five girls), four with methylation mosaicism, and two with size mosaicism. The Mullen Scales of Early Learning was administered to assess cognitive level in the FXS group (MSEL; Mullen, 1995). The MSEL is a developmental assessment standardized in children from birth to 68 months and consists of Gross Motor, Fine Motor, Receptive Language, Expressive Language, and Visual Reception subscales. To calculate the mental age of each FXS participant, subscale age equivalencies (excluding Gross Motor) were averaged and converted to months and days. Gross Motor scores are less valid in children over the age of 33 months and, given the wide age range of our participants, this subscale was excluded from the MA calculation (Mullen, 1995; Burris et al., 2017; Yoo et al., 2017).

Participants were excluded for failure to provide valid data on at least 20% of trials on the gap-overlap paradigm (i.e., children with recorded data for fewer than four trials in either gap or overlap conditions). The final FXS sample consisted of 18 children, chronological ages 7.25–68.02 months (*M* = 39, SD = 20.5), mental ages 5.15–44.23 months (*M* = 22.9, SD = 10.3); 14 males, four females. To control for cognitive level and overall development, two typically developing (TD) comparison groups were recruited. A group was matched to the mental age (MA) of the FXS sample, and consisted of 20 young children, chronologically aged 5.23–55.05 months (*M* = 23, SD = 13); 11 males, nine females. The FXS and MA groups’ mental ages were not statistically different (*t* (36) = −0.088, *p* = 0.6). A second group was closely matched to the chronological age (CA) of the FXS sample, and consisted of 20 young children, chronologically aged 6.3–68.07 months (*M* = 40.2, SD = 18.5); 14 males, six females. The FXS and CA groups’ chronological ages were not statistically different from one another (*t* (36) = −0.202, *p* = 0.84). See Table 1 for a breakdown of ages and number of usable trials across the three groups. The Institutional Review Board of the University of California, Davis, approved the experimental protocol, and informed consent was obtained from a parent or caregiver of each participant.

**Table 1:** The mean chronological age, mental age, and number of valid trials for each developmental group.

	FXS	TD-MA	TD-CA
*N*	18 (4 females)	20 (9 females)	20 (6 females)
Chronological age *M* (SD)	38.9 (20.5)	23.03 (12.8)	40.2 (18.5)
Chronological age range	7.25–68.02	5.23–55.05	6.3–68.07
Mental age *M* (SD)	22.9 (10.3)	N/A	N/A
Mental age range	5.15–44.23	N/A	N/A
Number of valid gap trials	171	170	178
Number of valid overlap trials	245	255	250

*Age is presented in months.days*.

### Apparatus

During the task, stimuli were presented on a 17-inch Tobii 1,750 LCD binocular infrared eye tracker with a screen resolution of 1,280 pixels × 1,024 pixels and a sampling rate of 50 Hz (Tobii Technology, Sweden). Tobii ClearView software was used to display stimuli and record gaze data, including a five-point calibration procedure. Children’s viewing distance from the eye tracker was approximately 60 cm and precision of eye coordinates was about 0.5° with 25–35 ms average accuracy in timing. Missing data due to blinks were interpolated, and gaze from at least one eye was used to determine gaze coordinates.

### Task Design

**Figure 1:**
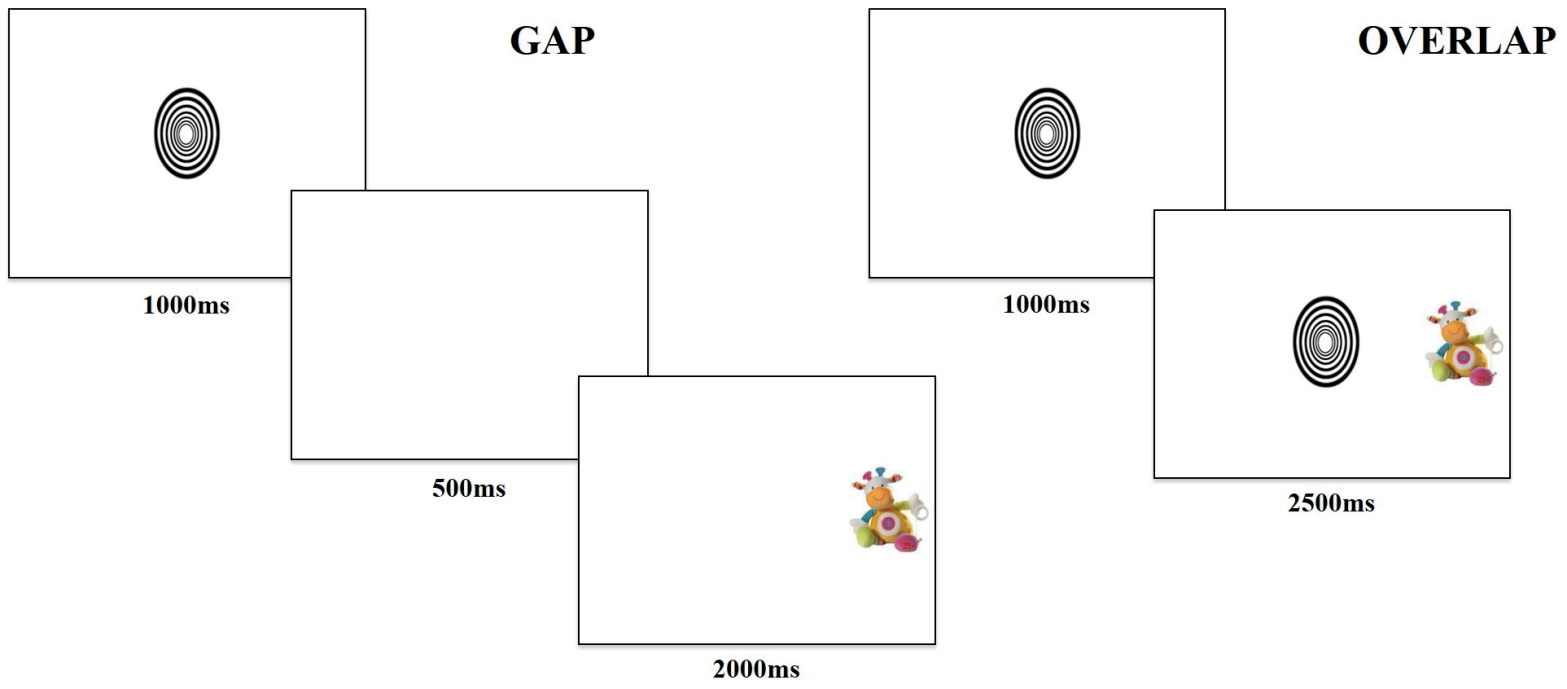
Example of gap and overlap trials.

The task contained two trial conditions, “gap” and “overlap,” with each type of trial lasting 3,500 ms. Gap and overlap trials (Figure 1) were each made up of two phases; phase one (lasting 1,000 ms) presented a central fixation object and phase two (lasting 2,500 ms) presented a target object. During phase one of the gap condition, the central object disappeared after 1,000 ms, then phase two began with a 500-ms “gap,” followed by presentation of the target object for 2,000 ms. In phase one of the overlap condition, the central object remained for the entire 1,000 ms, followed by phase two, where the central object persisted such that the target and central object “overlapped” for 2,500 ms by occupying adjacent positions on the screen.

The task consisted of 36 trials, counterbalanced between 18 gap (nine with target on the left) and 18 overlap (nine with target on the left) conditions. Participants saw a fixed-order randomized presentation of trials. The central fixation image fit into an ellipsis measuring 3×3 degrees of visual angle and cycled randomly through six different high-contrast black and white shapes. The peripheral target image fit into a rectangle measuring 3×5 degrees of visual angle and rotated through 18 different colorful pictures of stuffed animal toys. The design and temporal parameters were chosen based on literature reporting the use of the gap-overlap task with infants (Peltola et al., 2008, 2009).

### Procedure

All children were seated in their caregiver’s lap approximately 60 cm from the eye tracker monitor in a dimly lit and quiet room. Caregivers were asked to not interact with their child during the task presentation. The experiment began with a five-point calibration procedure, during which caregivers were asked to close their eyes to verify gaze data collected were from the child. The calibration routine was repeated until all five points were captured. There was then a continuous presentation of 36 trials, each lasting 3,500 ms, for a total presentation time of 126 s (2.1 min).

### Data Preparation and Analysis

Using ClearView Tobii analysis software, eye tracking data were analyzed with the Area-of-Interest (AOI) tool (Tobii Technologies, Sweden). AOIs were created separately by defining an area around the fixation and the target. The primary measure of interest was latency to first fixate to the target, where a fixation is defined as gaze within the AOI greater than 100-ms duration. Time of interest began as soon as the target appeared on screen and ended at the point of transition to the next frame. Valid trials were considered those on which: (1) the child did not look away from the screen at any point, and (2) gaze was on the central fixation prior to presentation of the peripheral stimulus. To be included in the analysis, children had to have seen at least four valid trials for each condition (>20% of trials). Exclusion criteria of four valid trials per condition were chosen based on prior research using the gap-overlap paradigm with infants (Peltola et al., 2008). There was no significant difference in number of valid trials by developmental group on gap trials (*F* (2, 51) = 0.710, *p* = 0.497) or overlap trials (*F* (2, 52) = 1.102, *p* = 0.34) (Table 1). Outliers greater or less than three standard deviations from the mean were excluded. One participant in the TD-CA group was considered an outlier based on latency on gap trials and removed from subsequent analyses. Latency to orient attention to the target was calculated by averaging the elapsed time to first fixate on the target for gap and overlap conditions separately for each developmental group. Using non-parametric tests, the gap effect was calculated as the difference in both average and median latency to fixate to gap and overlap conditions for each developmental group. Finally, given the previously documented developmental progression of the gap effect, we explored the impact of chronological and mental age (FXS only) on average latency to the gap effect.

## Results

### Gap Effect

**Figure 2:**
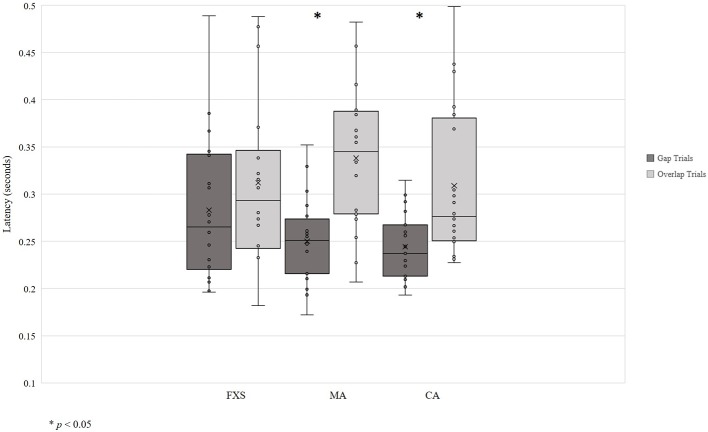
Saccadic reaction time in seconds. *p < 0.05.

Data visualization showed unequal variance across developmental groups, so homogeneity of variance was assessed using Levene’s Test of Equality of Error Variances. Levene’s test was significant for gap trials [*F* (2, 54) = 5.76, *p* = 0.005], indicating that equality of variances across developmental groups could not be assumed (Figure 2). Thus, due to the small sample size and non-normal data distributions, non-parametric tests were used to evaluate the gap effect. Specifically, the Wilcoxon matched-pairs signed-ranks test for pairwise comparisons of within-group responses across gap and overlap conditions was used.

Results show the expected gap effect in both TD-CA and TD-MA groups: using average latency, participants were slower to orient to peripheral targets on overlap trials than on gap trials (TD-CA: gap = 0.24 s; overlap = 0.31 s; *z* = −2.93, *p* = 0.03; TD-MA: gap = 0.25 s; overlap = 0.34 s; *z* = −3.55, *p* = 0.00). There was no significant gap effect in the FXS group (gap = 0.28 s; overlap = 0.31 s; *z* = −1.28, *p* = 0.20), indicating that children with FXS shift attention to the peripheral target at the same speed regardless of the presence or absence of a central stimulus. Analyses using median latency to gap and overlap trials revealed the same pattern of a significant gap effect for both TD-CA (*z* = −2.98, *p* = 0.003) and TD- MA groups (*z* = −3.51, *p* = 0.000), but no significant gap effect in the FXS group (*z* = −1.54, *p* = 0.127).

**Figure 3:**
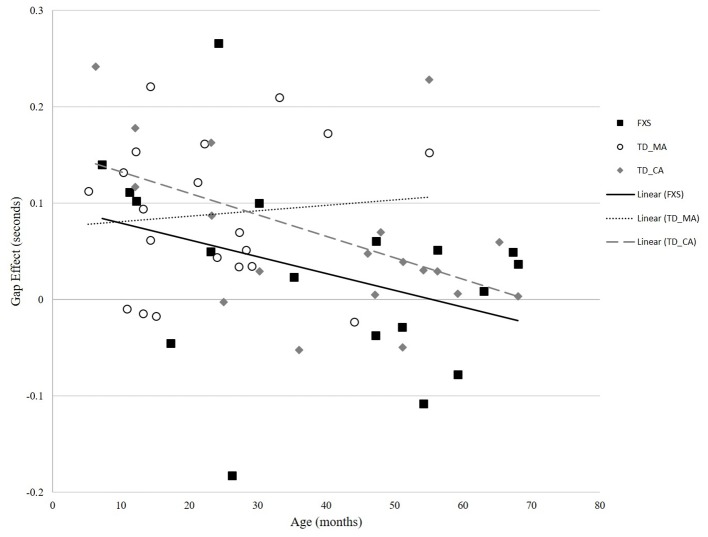
Correlation between age and gap effect across FXS and TD groups.

In the TD-MA group, there was no significant correlation between age and gap effect (*r* = 0.094, *p* = 0.693) (Figure 3). In the TD-CA group, there was a significant correlation between age and gap effect (*r* = −0.497, *p* = 0.031), such that gap effect latency declined with increasing age. In the FXS group, there was a significant correlation between mental age and gap effect (*r* = −0.584, *p* = 0.011), and chronological age and gap effect (*r* = −0.591, *p* = 0.010), reflecting a reduced gap effect latency with increasing chronological age, as seen in the TD-CA group.

## Discussion

The present study investigated attention orienting in young children with FXS and typically developing chronological and mental age-matched children. We used eye tracking methodology with a gap-overlap task to calculate the gap effect, a measure of visual orienting. The results of the present study replicate prior research with typically developing children, showing a faster saccadic latency on gap conditions than on overlap conditions (gap effect) in both chronological and mental age-matched groups. Thus, we can conclude that our task parameters were sufficient to elicit competition between the central stimulus and peripheral target, requiring attentional disengagement from the central stimulus in the overlap condition. Further, there was an age-related decline in latency on the gap effect in TD-CA and FXS groups, but not the TD-MA group, reflecting increasing maturity of the visual system. In the FXS group, despite a normative pattern of reduced latency on the gap effect with increasing age, and unlike both TD groups, saccadic latencies between gap and overlap conditions were not significantly different, indicating no gap effect. This finding suggests that attention engagement is impaired in FXS and lends behavioral support to our broader hypothesis that visual processing deficits in FXS are rooted in fronto-parietal dysfunction.

### Absent Gap Effect in Fragile X Syndrome

The latency to fixate to gap and overlap trials was not statistically significantly different in the FXS group, resulting in an absent gap effect that is otherwise present in both the typically developing chronological and mental age-matched groups. Based on prior literature, we contend that the gap-overlap paradigm taps into subcortical mechanisms on gap conditions, while fronto-parietal mechanisms are necessary for attention orienting on overlap conditions. The absence of a gap effect suggests that these two conditions are not processed differentially in the FXS group. During overlap trials, the visual system requires disengagement from the central fixation to orient to the peripheral target. However, given that we see no difference in performance on gap and overlap conditions, we interpret this finding as evidence for overall reduced attentional engagement, driven by a dysfunctional parietal network. Importantly, posterior parietal orienting mechanisms are necessary for inhibiting reflexive saccades, and rely on the subcortical collicular orienting pathway, both of which are impacted in FXS. As such, when reflexive saccades are not inhibited by the PPC and frontal regions (therefore, reduced attentional engagement to the central fixation), saccades to the peripheral target are not affected by the presence of the central fixation, resulting in faster latencies.

Based on the results of the current study, in conjunction with other findings in the FXS literature, we contend that disrupted visual orienting behavior may be one area driving atypical developmental outcomes in FXS. It is well established that the specific visuospatial and dynamic processing deficits noted in older individuals with FXS involve attention-mediated mechanisms supported by dorsal stream processing and the PPC (Scerif et al., 2005; Farzin and Rivera, 2010). The present findings suggest that there is also a basic impairment in orienting visual attention in order to fixate to static stimuli. Quick, uninhibited saccades exhibited by the FXS group in the current task suggest reduced attentional engagement, and could result in less time allocated to processing static stimuli, leading to important information being “missed” in a real-world environment. According to neurodevelopmental theories of attention and learning, impaired attention orienting could negatively impact basic statistical learning mechanisms typically operational in infancy and early development (Baker et al., 2004; Arciuli, 2017). In turn, these effects have the potential to developmentally cascade into an abnormal cognitive behavioral phenotype, such as the one seen in FXS.

It remains to be tested whether attentional orienting measures in the gap-overlap paradigm can be linked to other measures of attention-mediated visual processing, in either TD or FXS populations. Future studies should address visual orienting behavior involved in more complex cognitive abilities (e.g., spatial indexing, multiple object processing). Further, while the role of FMRP in development of visual circuits is not fully understood, there is some preliminary evidence to suggest that absence of the protein impacts development and function in the superior colliculus, a pathway key in visual orienting and the PPC (Kay et al., 2018). As such, deficits in visual attention and orienting in FXS may, in part, stem from disrupted production of FMRP, impacting both atypical subcortical and cortical development. The present study included predominately full mutation FXS subjects, limiting variability in FMRP expression, and was therefore unable to directly test this point. Future research should address this question by measuring variability in *FMR1* gene expression and subsequent development and connectivity of visual circuitry in both subcortical and cortical structures. Finally, although the current study included a relatively small sample size, the present findings highlight the utility of infrared eye tracking methodology in measuring visual attention in this population, and provide a solid foundation for further investigation.

The present study adds to the literature by documenting disrupted attentional engagement in young children with FXS. Overall, the findings bolster the general notion of parietal dysfunction in FXS, and further support the specific characterization of FXS as a disorder involving early and basic attention-mediated visual processing deficits.

## Ethics Statement

This study was carried out in accordance with the recommendations of Institutional Review Board of the University of California, Davis. Written informed consent was obtained from all parents or legal guardians on behalf of the child. All subjects gave written informed consent in accordance with the Declaration of Helsinki. The protocol was approved by the UC Davis Social and Behavioral Committee C.

## Author Contributions

EO and SR contributed to the conception and design of the study. MC, JB, and EO completed data collection and performed the statistical analysis. MC, JB, EO, and SR co-wrote the manuscript. All authors contributed to manuscript revision, read and approved the submitted version.

## Conflict of Interest Statement

The authors declare that the research was conducted in the absence of any commercial or financial relationships that could be construed as a potential conflict of interest.
